# Electronic Structure and Photoactivity of Organoarsenic Hybrid Polyoxometalates

**DOI:** 10.1021/acs.inorgchem.2c04249

**Published:** 2023-02-10

**Authors:** Alexander J. Kibler, Nicole Tsang, Max Winslow, Stephen P. Argent, Hon Wai Lam, David Robinson, Graham N. Newton

**Affiliations:** † The GSK Carbon Neutral Laboratories for Sustainable Chemistry, School of Chemistry, University of Nottingham, Jubilee Campus, Nottingham NG7 2TU, U.K.; ‡ School of Chemistry, University of Nottingham, University Park, Nottingham NG7 2RD, U.K.; § Department of Chemistry and Forensics, School of Science and Technology, Nottingham Trent University, Clifton Lane, Nottingham NG11 8NS, U.K.

## Abstract

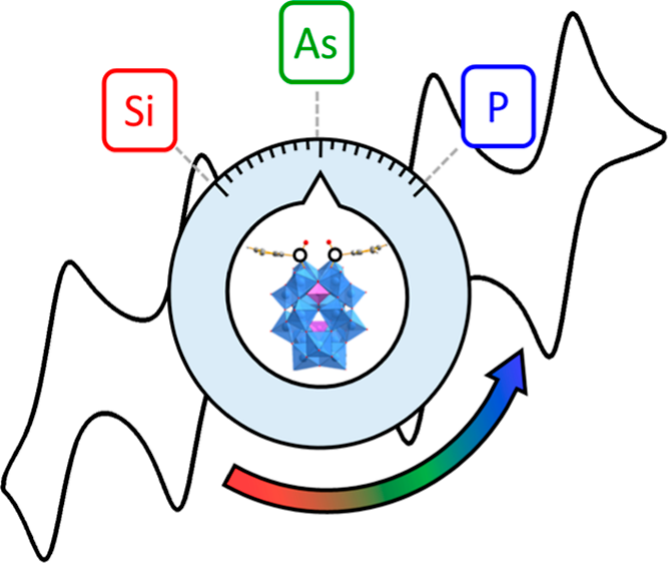

Organofunctionalization of polyoxometalates (POMs) allows the preparation of hybrid molecular systems with tunable electronic properties. Currently, there are only a handful of approaches that allow for the fine-tuning of POM frontier molecular orbitals in a predictable manner. Herein, we demonstrate a new functionalization method for the Wells–Dawson polyoxotungstate [P_2_W_18_O_62_]^6–^ using arylarsonic acids which enables modulation of the redox and photochemical properties. Arylarsonic groups facilitate orbital mixing between the organic and inorganic moieties, and the nature of the organic substituents significantly impacts the redox potentials of the POM core. The photochemical response of the hybrid POMs correlates with their computed and experimentally estimated lowest unoccupied molecular orbital energies, and the arylarsonic hybrids are found to exhibit increased visible light photosensitivity comparable with that of arylphosphonic analogues. Arylarsonic hybridization offers a route to stable and tunable organic–inorganic hybrid systems for a range of redox and photochemical applications.

## Abstract 2

Organoarsonate functionalization offers a new route to tune the structure and electronic physical properties of polyoxometalates. Here, we demonstrate the opportunities offered by this new approach to cluster hybridization.

## Introduction

Polyoxometalates (POMs) lie at the interface between oxoanions and solid-state metal oxides. Typically comprising group 5 and 6 metals in their highest oxidation states, they exhibit excellent thermal and chemical stability,^1,2^ photosensitivity,^3,4^ tunable solubility,^5^ and the capacity to undergo reversible, multielectron redox processes.^6,7^ These characteristics have led to their application in fields such as catalysis,^8,9^ energy storage,^8,9^ medicine,^10^ non-linear optics,^11^ and data processing.^12^

In recent years, the covalent functionalization of POMs with organic groups, forming the so-called organic–inorganic hybrid POMs, has emerged as a powerful tool for the modulation of their physical and electronic properties.^13^ As opposed to organic cation modification, covalent functionalization offers a direct and robust method for intimately controlling POM properties while installing the functionality in a controlled and modular manner. This is particularly salient in the context of molecular orbital engineering where the rational design of species with well-defined electronic properties remains a key target for both catalytic and photochemical applications.^14,15^

Fine-tuning the electronic properties of hybrid POMs through variation of the organic substituent(s) requires sufficient electronic communication between the inorganic and organic moieties. This is achieved through the judicious choice of the POM-organic anchor unit.^16^ The most prominent examples in which POM properties are dependent on the nature of the organic component include organoimido-functionalized molybdates and organophosphonic modified tungstates.^17,18^ Although the former method has been used prolifically for Lindqvist polyoxomolybdates ([Mo_6_O_19_]^2–^), the use of organophosphonates to tune the properties of multiredox active POMs such as the Keggin and Wells–Dawson polyoxotungstates is underexplored.^19−21^ A leading example of organophosphonic functionalization with Wells–Dawson phosphotungstates was published by Fujimoto et al. as a method for photoactivation without the need for photosensitizing moieties.^17^ In this work, computational modeling and experimental results demonstrated that by altering the electronic properties of the arylphosphonate ligands grafted onto the POM, the frontier MO energies of the POM, and hence the redox properties, could be rationally modified. This effect was also demonstrated in photocatalytic studies, where the POM hybrids were significantly more active for the oxidation of indigo dye than the plenary K_6_[P_2_W_18_O_62_] starting material, and there was a clear relationship between photoactivity and the electron-withdrawing character of the arylphosphonate.^17^

Despite this achievement and the abundant opportunities that this methodology offers, the development of these types of organic–inorganic hybrid POMs is still underexplored. A recurring issue for organophosphonic derivatives is that their lowered lowest unoccupied molecular orbital (LUMO) energy stymies their aerobic reoxidation, which hampers their appeal as photocatalysts.^22^ Key to advancing this area is the development of other functionalization approaches expanding the opportunities for bespoke hybrid POM systems. Phenylarsonic acids are isoelectronic to phenylphosphonic acids yet have not been explored as electronic modulators of redox-rich tungstate-based POMs. In fact, only a small number of examples exist where polyoxotungstates have been functionalized with organoarsenic groups, and these have mainly served as structural motifs for accessing new cluster morphologies.^23−31^

Here, we report the synthesis and structural and electronic characterization of a new family of arylarsonic-functionalized Wells–Dawson polyoxotungstates with the formula [P_2_W_17_O_61_(*p*-AsOC_6_H_4_R)_2_]^6–^, where R = H, NH_2_, and NO_2_. We demonstrate through computational and electrochemical studies that the arylarsonic functionality allows for the fine-tuning of the POM frontier molecular orbitals. We also demonstrate that the arylarsonic moiety photoactivates the POM toward visible light by lowering the LUMO energy, highlighting that organoarsonic hybridization could be a route toward high-performance POM hybrid photocatalysts.

## Results and Discussion

**Figure 1:**
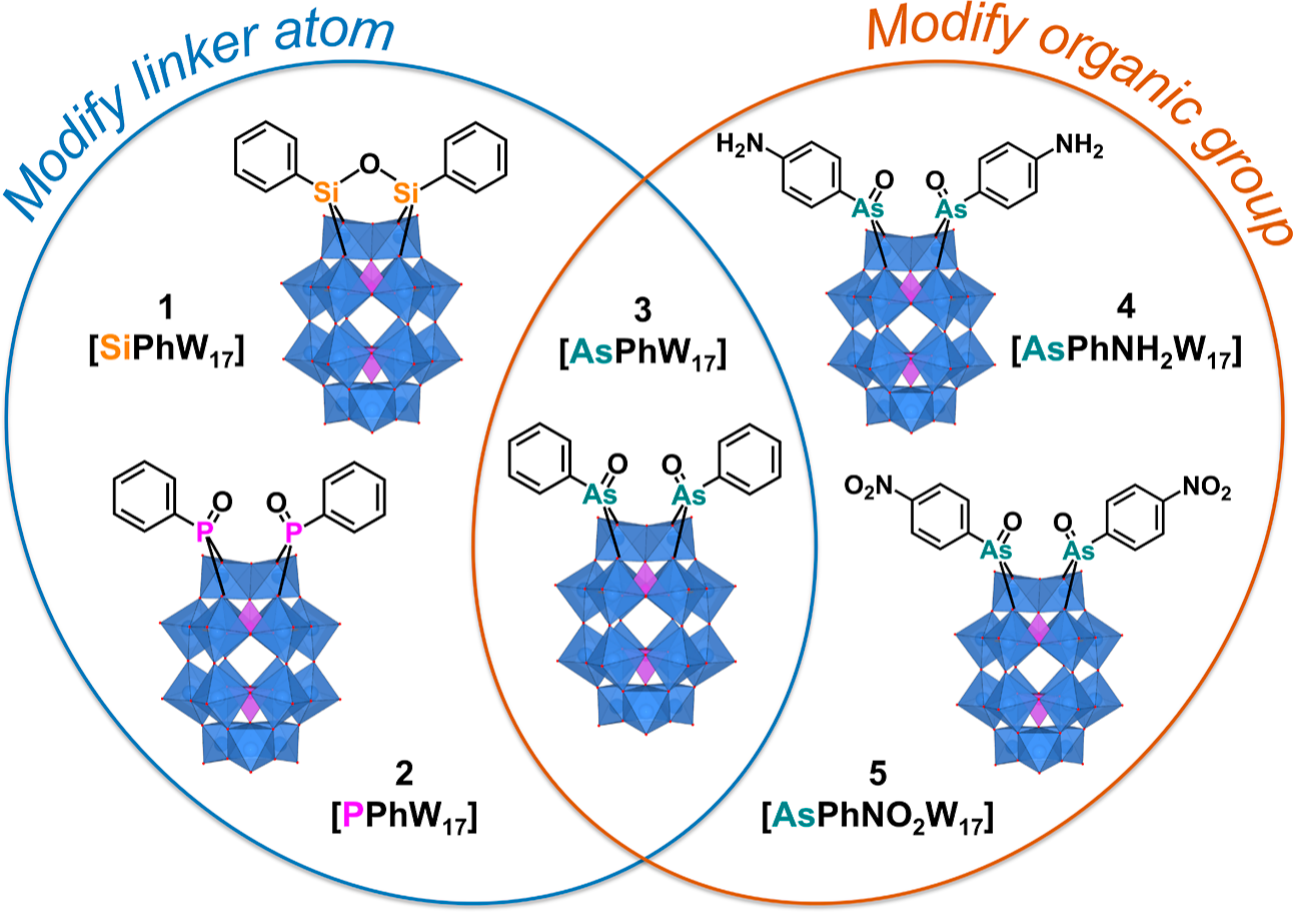
Molecular representation of structurally related compounds **1**–**5** showing the structure of the POM core and the connectivity of the organic groups. Color code: blue octahedral: “WO_6_” and purple tetrahedral: “PO_4_”.

To explore the impact of arylarsonic groups on the Wells–Dawson POM, the phenylarsonic acid hybrid [P_2_W_17_O_61_(AsOC_6_H_5_)_2_]^6–^ (**3**) was synthesized and compared to previously reported phenylsiloxane [P_2_W_17_O_62_(SiC_6_H_5_)_2_]^6–^ (**1**) and phenylphosphonic [P_2_W_17_O_61_(POC_6_H_5_)_2_]^6–^ (**2**) hybrids.^19^ In addition, to probe the degree of orbital fine-tuning that could be achieved through modifications to the aromatic ring, we synthesized phenylarsonic analogues containing an electron-donating amino group [P_2_W_17_O_61_(AsOC_6_H_4_-*p*-NH_2_)_2_]^6–^ (**4**) or electron-withdrawing nitro group [P_2_W_17_O_61_(AsOC_6_H_4_-*p*-NO_2_)_2_]^6–^ (**5**) at the *para*-positions (Figure 1). The compounds were isolated as both their *n*-tetraethylammonium (TEA) and *n*-tetrabutylammonium (TBA) salts.

### Synthesis and Structural Characterization

**Figure 2:**
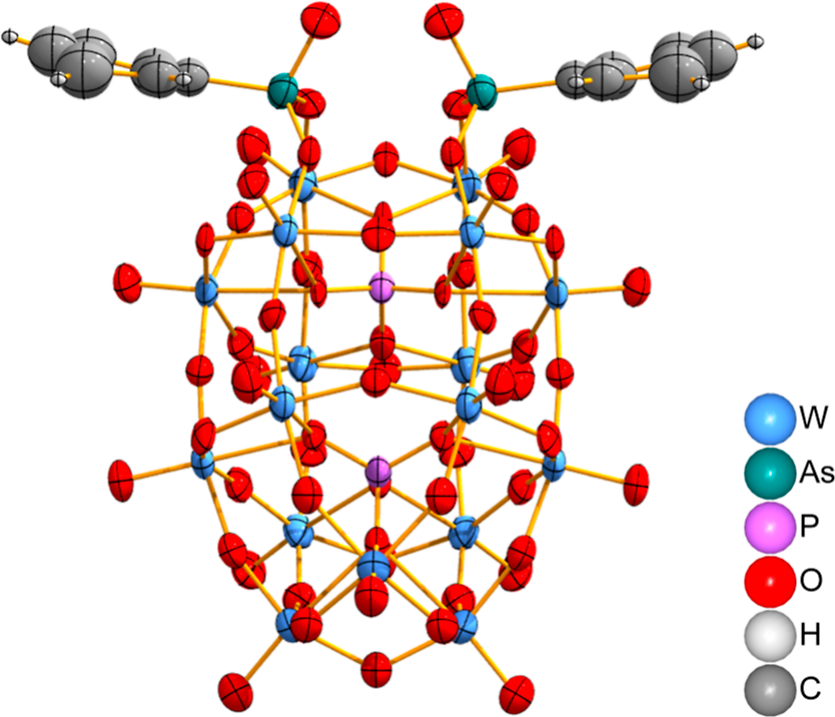
X-ray crystal structure of **3** with TEA cations omitted for clarity (50% thermal ellipsoid probability). Atom color key in the bottom right corner.

Compounds **3**, **4**, and **5** were synthesized using a modified literature procedure.^17^ Acid-catalyzed condensation of phenylarsonic acid, *p*-arsanilic acid, or *p*-nitarsone with the lacunary potassium Wells–Dawson POM K_10_[P_2_W_17_O_61_] in DMF followed by the addition of TEABr or TBABr yielded the desired compounds as either off-white (**3**, **5**) or dark orange (**4**) solids in 66, 71, and 45% yields, respectively (TEA salts). Each compound was characterized by ^1^H and ^31^P NMR spectroscopy, FTIR spectroscopy, and ESI-MS (see Methods, Figures S1–S5, and Tables S1–S5 in the Supporting Information). Additionally, single crystals of **3** were obtained from vapor diffusion of EtOAc into a solution of **3** in DMSO, and X-ray crystallographic analysis was performed (Figure 2). Compound **3** crystallizes in the orthorhombic crystal with space group *Pnma*. Two TEA cations were modeled in the asymmetric unit, but other residual electron densities from the disordered solvent and cations were treated with PLATON SQUEEZE.^32^ Structurally, the two acidic groups of the phenylarsonic acids have condensed with two basic oxo sites of the lacuna, analogous to structures reported for organophosphorus Wells–Dawson hybrids.^17,33^ Although the local geometry is highly reminiscent of that seen in the reported phenylphosphonic hybrid POM crystal structure,^17^ the phenylarsonic derivative possesses longer bond lengths between the arsenic atom and the aromatic carbon (As–C = 1.90(2) Å and P–C = 1.79(2) Å) and POM oxygen atoms (As–O = 1.68(1) and 1.70(2) Å and P–O = 1.54(1) Å) as a result of poorer orbital overlap with the heavier arsenic atom. These bond lengths are similar to those seen in the recently reported phenylarsonic hybrid of the macrocyclic “P_8_W_48_” POM (Table S7).^28^

### Electronic Characterization

**Figure 3:**
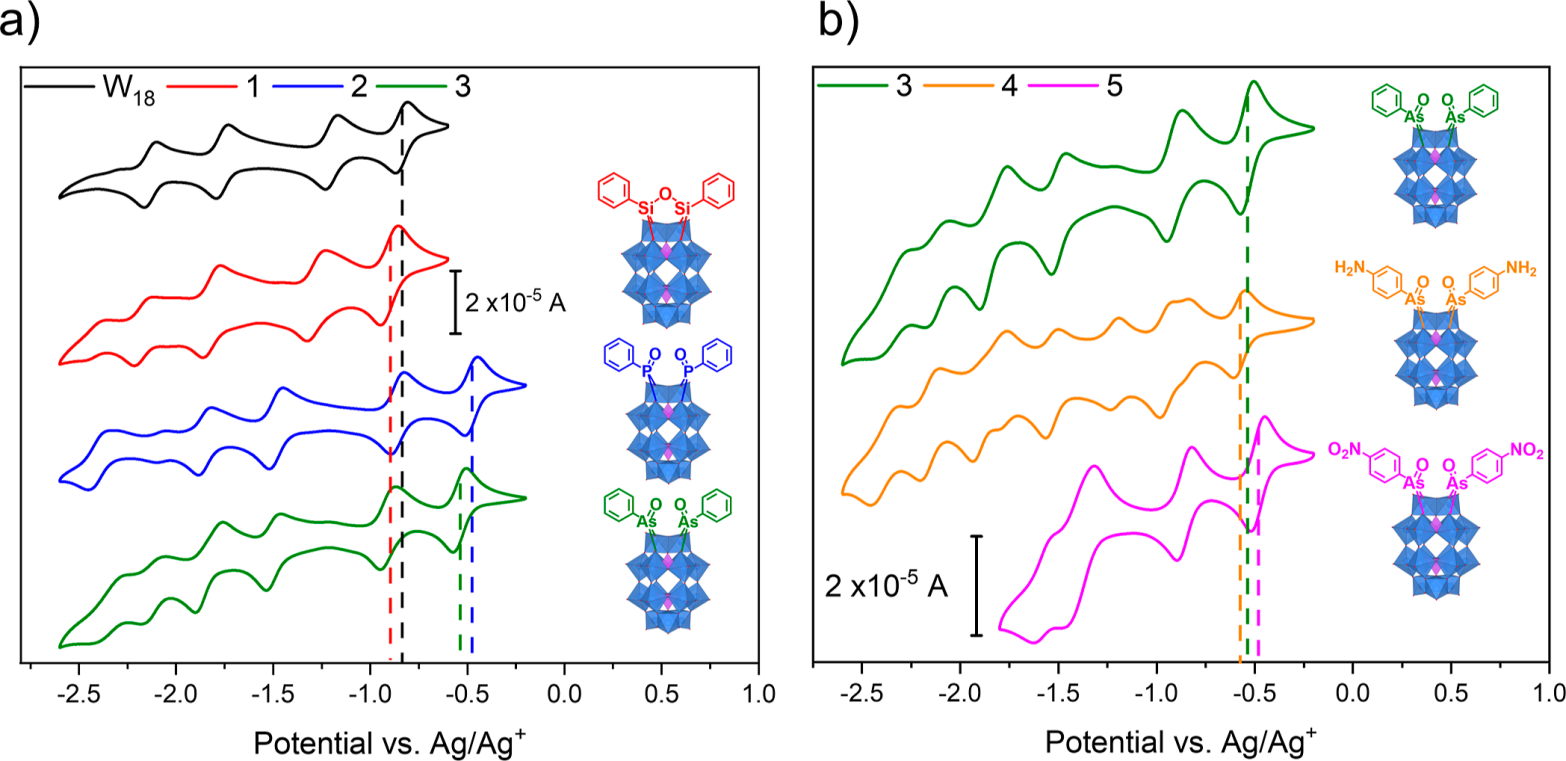
CVs of 1 mM POM in acetonitrile with 0.1 M TBA[PF_6_] as the supporting electrolyte. Recorded using a GC working electrode (0.071 cm^2^), Pt wire counter electrode, and Ag^+^|Ag reference electrode. All CVs were recorded at 0.1 V s^–1^, and the third cycle is plotted. (a) Relationship between phenyl derivatives with different linkers and the plenary POM. (b) Relationship between the as-hybridized POMs bearing different aryl groups.

The impact of hybridization on the electronic properties of the POMs was investigated using cyclic voltammetry. Measurements were performed in anhydrous MeCN with TBA[PF_6_] (0.1 M) as the supporting electrolyte under an inert atmosphere. The electrochemical setup employed a glassy carbon working electrode (*d* = 3 mm and *A* = 0.071 cm^2^), platinum counter electrode, and a AgNO_3_|Ag non-aqueous reference electrode, and samples were measured at 1 mM concentration. First, we compared the electrochemistry of the plenary parent POM TBA_6_[P_2_W_18_O_62_] (“W_18_”) with the literature-reported organosilicon (**1**) and organophosphorus (**2**) derivatives and the isostructural organoarsonic compound (**3**). The voltammetry of each showed the characteristic multielectron reduction behavior of Wells–Dawson POMs. As previously described, employing organosilicon groups results in a negative shift in the first reduction potential compared to the plenary Wells–Dawson POM, whereas organophosphorus groups give a positive shift, due to the ability of the linker to control the degree of orbital overlap.^19^Figure 3a and Table 1 demonstrate that the redox properties of **3** are closely aligned to the isoelectronic phenylphosphonic derivative **2**; however, the first reduction potential is not as positively shifted. This is likely a result of the longer bond lengths and hence poorer orbital overlap, with arsenic and its neighboring atoms. The degree of redox tunability of organoarsonic hybrids was then investigated by comparing the voltammetry of **3** with structurally related derivatives bearing *p*-substituted electron-donating amino (**4**) or electron-withdrawing nitro (**5**) groups (Figure 3b and Table 1). It is evident that the electronic character of the ring can be modulated to tune the redox properties of the hybrid, with the first redox potentials of the different systems following the expected trend, shifting positively as the electron deficiency of the ring is increased.

**Table 1:** Electrochemical Parameters, *E*_1/2_, Δ*E*_p_, and *i*_p,a_/*i*_p,c_, for the First Redox Couple for Each POM (1 mM) Obtained from CVs at 0.1 V s^–1^

POM	first reduction potential (*E*_1/2_, V)	Δ*E*_p_ (mV)	*i*_p,a_/*i*_p,c_
W_18_	–0.86	65	1.05
**1**	–0.90	94	1.22
**2**	–0.48	63	1.02
**3**	–0.54	63	1.37
**4**	–0.58	60	1.22
**5**	–0.49	69	1.10

Beyond the first redox potential, the voltammetry of each compound showed the characteristic multielectron reduction behavior of Wells–Dawson POMs. The phenylarsonic hybrid (**3**) shows a similar voltametric profile to the phenylphosphonic analogue (**2**) with the first four chemically reversible processes all falling within 100 mV of the corresponding processes in the voltammogram of **2**. In all cases, the peak-to-peak separation of the processes become slightly larger at more negative potentials but remain in the range of 60–150 mV. The arylarsonic hybrids bearing functional groups (**4** and **5**) exhibit differing redox behavior. The amino derivative **4** possesses two additional redox processes (*E*_1/2_ = −0.84 and −1.22 V) while retaining the six redox peaks observed in **3**. These extra reductions can be ascribed to proton-coupled redox processes originating from the presence of water in the sample.^34^^1^H NMR analysis confirmed the presence of significantly more water in the powder sample of **4** compared with **3** and **5**. This is likely a result of the hydrophilicity of the amine groups. The voltammetry of the nitro derivative **5** clearly shows a new multielectron reduction at −1.39 mV which can be credited to the concomitant reduction of the two nitro groups and is consistent with the previously reported reduction potentials for nitroarenes.^35^ No further processes were observed at negative potentials due to the onset of solvent degradation, probably catalyzed by the reactive nitro radical anions.

UV–vis spectroscopy was employed to probe the photochemical nature of the new hybrids. It has previously been described how adjusting the electronic character of phenylphosphonic hybrids results in observable trends in electrochemical behavior but minimal differences in the absorption maxima or tailing in the UV–vis spectrum.^17^Figure S7 shows that for compounds **1**–**5**, there are only minimal differences in the absorption edge, with the exception of **4**, which has an extended tailing into the visible region. The absorption profiles of **1**–**3** and W_18_ are nearly identical, which suggests that these are contributed solely by POM-based absorbances. In the UV region, we observe that **4** and **5** have discrete peaks at 258 nm (ε = 91,654) and 259 nm (ε = 64,582), respectively, which enhance their overall absorbance, likely resulting from the absorbance of the N-substituted aromatic rings. Overall, the substitution has a much less profound effect on the absorption properties than the redox characteristics.

### TD-DFT Calculations

**Figure 4:**
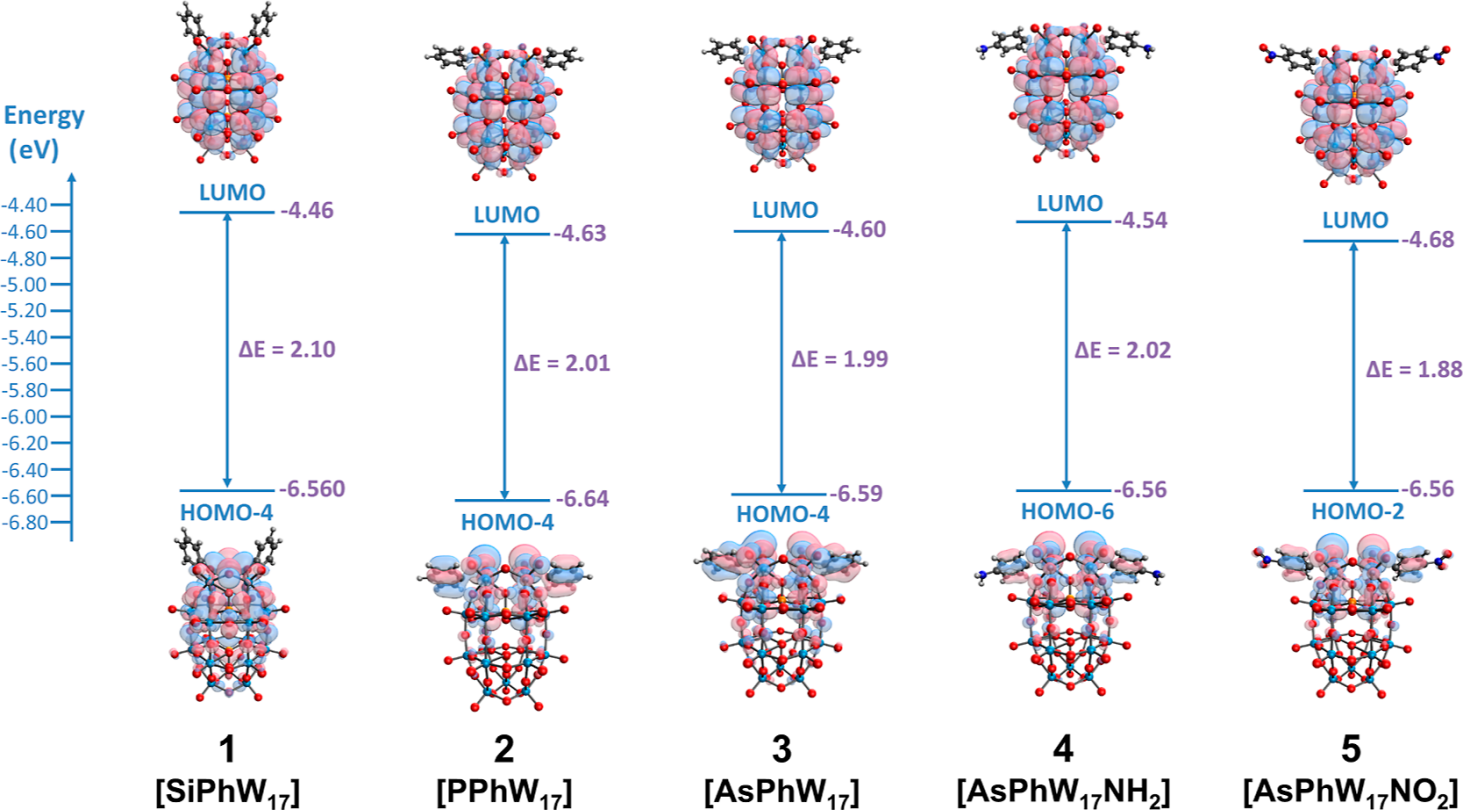
TD-DFT-calculated LUMO and HOMO-X (highest energy HOMO with POM orbital character) energies for the five hybrid compounds, showing the HOMO–LUMO gaps and orbital distributions. Trends in LUMO energy correlate well with reduction potentials obtained from CV experiments, and HOMO–LUMO gap differences reflect absorption edge values from UV–vis spectra.

To improve our understanding of the electronics of the hybrid POMs, we employed DFT to calculate the frontier molecular orbitals and examine the orbital distributions across the organic and inorganic moieties. DFT calculations were performed using geometry optimization with BP86/CRENBL, with solvation effects accounted for using the conductor-like polarizable continuum model (C-PCM). Figure 4 shows the calculated energies of the highest occupied molecular orbital (HOMO) and LUMO, the calculated HOMO–LUMO gap, and the orbitals for each of the hybrid structures. Here, we used only the frontier orbitals which contained appreciable POM character as we know that both POM redox reactions and photochemistry are localized on tungsten and oxo sites, respectively. The calculated energies for the LUMO energies are in good agreement with trends obtained from electrochemistry measurements. For example, **1** has both the highest LUMO energy (−4.46 eV) and the most negative first reduction potential (−0.90 V), whereas **2** and **5** have the lowest LUMO energies (−4.63 and −4.68 eV, respectively) and the most positive first reduction potentials (−0.48 and −0.49 V, respectively). Additionally, the orbital distribution on the HOMO shows that the organoarsonic derivatives **3–5** are similar to the phosphonic analogue, **2**, which all show orbital mixing between the organic and inorganic components, whereas the phenylsilyl derivative, **1**, has HOMO orbitals that are constrained to the POM core. We also observe that shifts in the LUMO are accompanied by similar shifts in the HOMO energies, which results in similar HOMO–LUMO gaps between the five derivatives—this matches our observation in UV–vis measurements, which shows minimal differences in the absorption edge regardless of the functionalization strategy.

### Photoreduction Studies

Encouraged by the experimental and theoretical results above, we were motivated to compare the photoactivity of the phenylarsonic hybrid **3** against the isostructural phenylsiloxane and phenylphosphonic acid hybrids **1** and **2**, respectively. We anticipated that **3** should be photoactive toward both UV and visible wavelengths, based on the stabilization of the LUMO energy and the computed smaller HOMO–LUMO gap.

**Figure 5:**
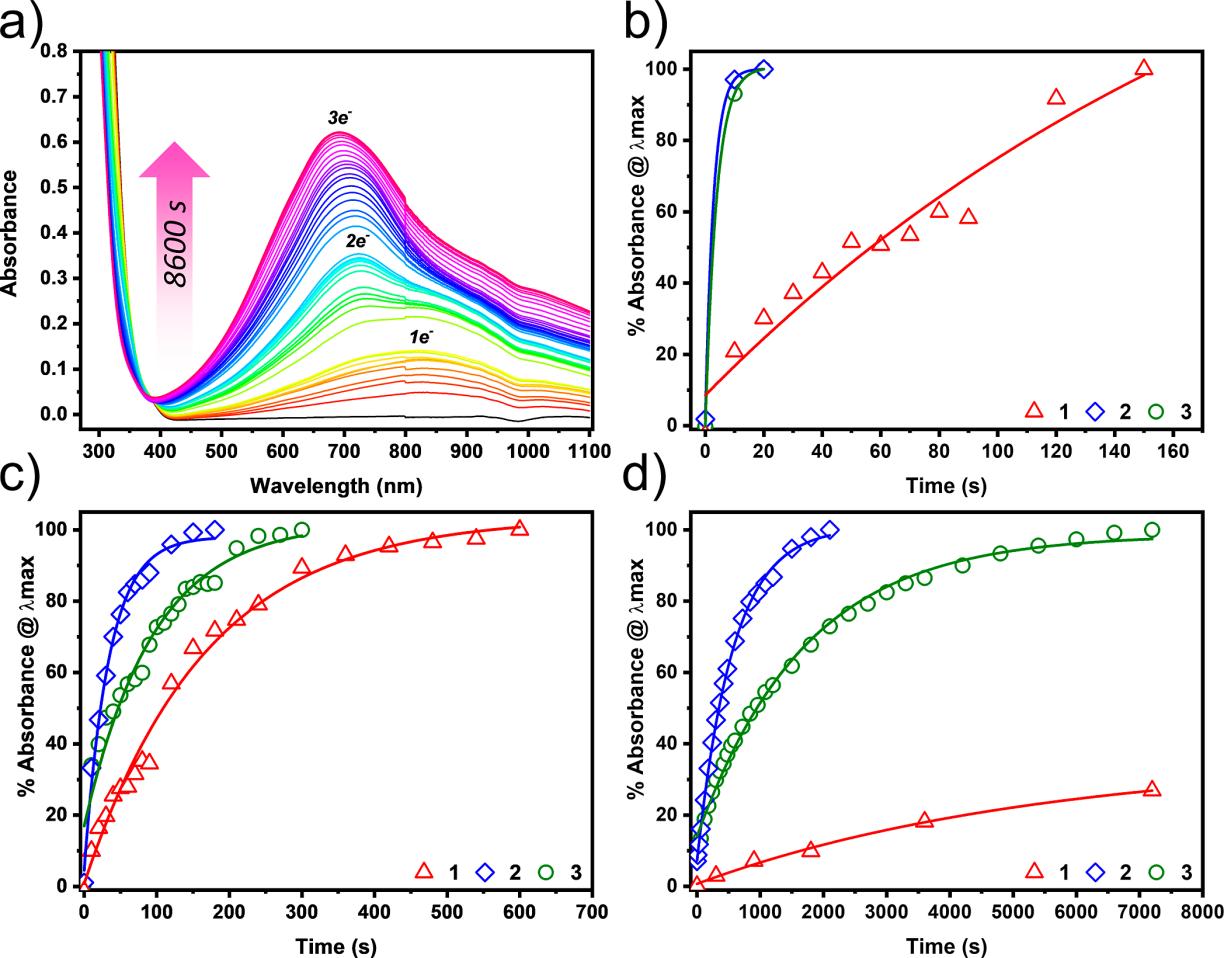
POM photoreduction studies in DMF (4 × 10^–5^ M POM) observing the rate at which the IVCT band saturates under photoirradiation: (a) UV–vis absorption spectrum showing the photoreduction of **1** in DMF over time without a filter. (b) Rate of saturation of the first IVCT band under broad-spectrum irradiation, (b) rate of saturation of the second reduced-state IVCT band under broad-spectrum irradiation, and (c) rate of saturation of the first reduced-state IVCT band under visible light irradiation (395 nm cutoff filter).

To this end, we performed the anaerobic photooxidation of DMF using **1**–**3**. Experimentally, we irradiated DMF solutions of the POM (40 μM) with a Hg(Xe) arc lamp both with and without a 395 nm cutoff filter. UV–vis analysis was simultaneously employed to measure the reduction of the POMs through the evolution of the intervalence charge transfer (IVCT) bands within the visible region of the spectrum. Each sequential reduced state of the POM gives rise to characteristic IVCT bands at different absorption maxima, which are convenient indicators for monitoring both the rate and extent to which the POM is reduced. This is exemplified in Figure 5a for **1** in the absence of a filter. All other spectra for **1**–**3** can be found in Figures S8–S12. Figure 5b–d shows the comparison of the rates at which the individual IVCT bands reach saturation for compounds **1**–**3** with and without a UV cutoff filter.

First, we measured the rate at which the POMs are photoreduced in DMF under broad-spectrum light without the use of a filter. We find that each of the POMs **1**–**3** undergo a one-electron reduction, manifesting as a band appearing between 780 and 840 nm (Figure 5a). For compounds **2** and **3**, this band saturates very quickly (<40 s), whereas for **1** (with a larger HOMO–LUMO gap), saturation of the band is considerably slower (150 s).

Each POM then undergoes a further reduction to the doubly reduced state, which gives a band at 725, 672, and 703 nm for compounds **1**–**3**, respectively. The rate at which this peak evolves differs substantially between the POMs (Figure 5b); compounds **2** and **3**, possessing the electron-withdrawing phenylphosphonic and phenylarsonic groups, respectively, saturate the fastest at 180 s and 300 s irradiation, respectively, followed by compound **1**, reaching saturation within 600 s.

We next explored the photoactivity of the hybrids toward visible light only using a 395 nm filter (Figure 5c). We found that under visible light irradiation, compounds **2** and **3** reached saturation of the 1e-reduced state within 2100 and 7200 s, respectively. In contrast, compound **1** reached only 27% saturation when the experiment was terminated at 7200 s.

In all cases, we observe that the IVCT evolution follows an exponential decay curve. This is due to the low concentrations of POM used and hence weak absorbance of the solution, which results in the reaction following first-order kinetics. The fitting of first-order kinetics is especially relevant for the UV cutoff filtered data as the initial absorbance is less than 0.1.^36^

These experiments signaled that functionalization through phenylphosphonic and phenylarsonic ligands gave compounds with enhanced photoactivity under both the broad spectrum and visible light. On completion of the experiments, the sealed cuvettes were exposed to air with no agitation which resulted in the slow bleaching of the solutions via aerobic oxidation. It was observed that the phenylarsonic derivative **3** decolored more rapidly than **2** over the course of 90 min, suggesting that phenylarsonic hybrid POMs may be of interest in photocatalysis where the rate of POM reoxidation is key to achieving rapid turnover.

## Conclusions

We have developed a new functionalization strategy using arylarsonic acids for the hybridization of electron-rich Wells–Dawson POM clusters. This study shows through both computational and experimental methods that the frontier molecular orbitals of the hybrids can be finely tuned by modulating the electronic properties of the rings, while also introducing the functionality that alters the electron storage capabilities of the molecule. In addition, we demonstrate that the arylarsonic derivative displays the photosensitivity that has been previously described for analogous arylphosphonic hybrids. The design of highly tunable POM structures with visible light photoactivity is a key target in the development of the next generation of photocatalysts for organic transformations—a field that has been dominated by the high-energy (UV) irradiation of decatungstate catalysts. The arylarsonic functionality expands the synthetic toolbox available to POM chemists for the design of bespoke systems with well-defined electronic and photophysical properties toward applications in photocatalysis and reversible photochromic devices.

## Supporting Information Available

Instrument and procedural details, experimental procedures including structural characterization, ESI-MS data including assignment tables, crystallographic details, and parameters, and UV–vis spectroscopy figures (PDF)
