# Supplementary material for: Electronic Structure and Photoactivity of Organoarsenic Hybrid Polyoxometalates
Source: Inorg Chem. 2023 Feb 10;62(8):3585–91. doi: 10.1021/acs.inorgchem.2c04249 (PMC9976276; doi:10.1021/acs.inorgchem.2c04249)
Supplement: Supplementary file 1 — ic2c04249_si_001.pdf [file ic2c04249_si_001.pdf]

Supporting Information

The Electronic Structure and Photoactivity of Organoarsenic Hybrid Polyoxometalates

Alexander J. Kibler,[a] Nicole Tsang,[a] Max Winslow,[c] Stephen P. Argent,[b] Hon Wai Lam,[a] David Robinson[c] and Graham N. Newton[a]*

[a]The GSK Carbon Neutral Laboratories for Sustainable Chemistry, School of Chemistry, University of Nottingham, Jubilee Campus, Nottingham, NG7 2TU, U.K.

[b]School of Chemistry, University of Nottingham, University Park, Nottingham, NG7 2RD, U.K.

[c]Department of Chemistry and Forensics, School of Science and Technology, Nottingham Trent University, Clifton Lane, Nottingham NG11 8NS, U.K.

Methods

NMR spectra were recorded on a Brucker DPX500 500 MHz nuclear magnetic resonance spectrometer and referenced to the residual NMR solvent signals. Mass spectra were recorded on a Bruker MicroTOF spectrometer by electrospray ionisation (ESI). ATR FT-IR measurements were performed on a Bruker Alpha Series FT-IR spectrometer equipped with an attenuated total reflectance (ATR) module. UV-vis spectroscopy of all samples were performed on a Cary UVVis NIR Spectrometer. Cyclic voltammetry measurements were performed using a CHI Instruments electrochemical workstation equipped with a standard three-electrode arrangement; working electrode: glassy carbon (d = 3 mm), counter electrode: Pt wire, reference electrode: Ag^+|Ag nonaqueous reference electrode. Measurements were performed in anhydrous dimethylformamide (DMF) using n-tetrabutylammonium hexafluorophosphate ($TBAPF_6$) (0.1 M) as a supporting electrolyte and 0.5 mM analyte. All solutions were purged with argon for at least 30 mins to remove O_2 and kept under a positive pressure of argon whilst performing the experiment. DFT calculations were performed using DFT calculations were performed using the BP86 functional[1] with the CRENBL basis set[2] and effective core potential used to model the core electrons. Solvation effects were accounted for using the polarizable continuum model (PCM). Calculations were performed with the Q-Chem software.[3] Photoreduction studies were performed under inert conditions in degassed DMF (2.5 mL) at 4 x 10^{-5} M concentration, samples were irradiated with a 300 W Xe Cermax lamp (operating at 200 W) either with or without a 395 nm cut-off filter. Aerobic oxidation measurements were performed by opening the system to atmosphere and taking UV-vis absorption spectra at 90 second intervals.

Synthesis and Characterization

All reagent were purchased from commercial suppliers and used without further purification. All solvents were reagent grade and used as received unless otherwise stated. $K_6[P_2W_{18}O_{62}]$, $K_{10}[P_2W_{17}O_{61}]$, $(TBA)_4[W_{10}O_{32}]$, $(TBA)_6[P_2W_{17}O_{62}(SiC_6H_5)_2]$ (**6**), $(TBA)_6[P_2W_{17}O_{63}(PC_6H_5)_2]$ (**7**) were prepared according to literature methods.

Synthesis of $(TEA)_6[P_2W_{17}O_{62}(SiC_6H_5)_2]$ (1)

1 was synthesised according to a modified literature procedure. Trimethoxysilane (37.3μL, 0.293mmol) was added to a mixture of methanol (15ml) and water (5ml). After stirring for 5

mins, $K_{10}[P_2W_{17}O_{61}]$ (0.5g, 0.110mmol) was added to the suspension and was stirred for a further 5 mins. The pH of the dispersion was then adjusted to 1.8 with 1M HCL causing the dissolution of the reagents and the development of a yellow solution. The solution was stirred for 30mins forming a white precipitate, which is removed by filtration. The filtrand is then stirred for 16hrs and the solvent is removed *in vacuo* to give a pale yellow powder (0.423g). The powder was redissolved in *ca.* 10ml water and to this was added N-tetraethylammonium bromide (0.273g, 1.30mmol) dissolved in 10ml of water with stirring, causing the immediate precipitation of a pale yellow solid. The suspension was stirred for 1hr and then filtered and washed with ethanol and diethyl ether, yielding the product as a pale yellow powder (0.287g, 57%). ^{1}H NMR (DMSO-d_6, 500MHz, ppm) δ 7.78-7.72 (m, 4H), 7.43-7.34 (m, 6H), 3.28 (q, J=7.2Hz, 48H), 1.21 (tt, J=7.3, 1.7Hz, 72H). ^{31}P NMR (DMSO-d_6, 500MHz, ppm) δ -10.76, -13.80. ATR-IR (cm^{-1}): 2980 (w,ν_{C-H}), 1482 (m,ν_{C-H}), 1391 (m,ν_{C-H}), 1085 (m, ν_{P-O}), 1128 (m, $\nu_{Si-O-Si}$), 1085 (m, ν_{P-O}), 1040 (s, ν_{Si-O-W}), 946 (s, $\nu_{W=O}$), 914 (s, ν_{P-O}), 790-741 (vs, ν_{W-O-W}), 697 (s, ν_{Si-C}).

Synthesis of $(TEA)_6[P_2W_{17}O_{63}(PC_6H_5)_2]$ (2)

2 was synthesised according to a modified literature procedure. Phenylphosphonic acid (39.5mg, 0.250mmol) was dissolved in DMF (15ml) with stirring to give a colourless solution. To this was added $K_{10}[P_2W_{17}O_{61}]$ (0.5g, 0.110mmol) followed by 12M HCl (49μL, 0.588mmol) giving a pale turbid solution. The solution was then heated at 90°C for 16hrs after which it had become a cloudy pale yellow suspension. This was cooled to room temperature and filtered to give a pale yellow-green solution. This was then added to a large excess of EtOAc and the suspension was centrifuged and the supernatant was discarded leaving a sticky green solid. The sticky green solid was repeatedly sonicated in CHCl3 until it became a free-flowing powder, which was collected by centrifugation and dried *in vacuo.* The green powder was then redissolved in *ca.* 10ml of water and to this was added N-tetraethylammonium bromide (0.273g, 1.30mmol) dissolved in 10ml of water with stirring, causing the immediate precipitation of a pale blue solid. The suspension was stirred for 1hr and then filtered and washed with ethanol and diethyl ether, yielding the product as a pale blue powder (0.381g, 76%). ^{1}H NMR (DMSO-d_6, 500MHz, ppm) δ 8.03-7.94 (m, 4H), 7.58-7.51 (m, 2H), 7.50-7.45 (m, 4H), 3.24 (q, J=7.2Hz, 48H), 1.19 (tt, J=7.3, 1.8Hz, 72H). ^{31}P NMR (DMSO-d_6, 500MHz,

ppm) δ 14.93, -11.32, -12.94. ATR-IR (cm^{-1}): 2957 (w,ν_{C-H}), 1484 (m,ν_{C-H}), 1392 (m,ν_{C-H}), 1135 (m, $\nu_{P=O}$) , 1087 (m, ν_{P-O}), 953 (s, $\nu_{W=O}$), 906 (s, ν_{P-O}), 731 (vs, ν_{W-O-W}).

Synthesis of $(TEA)_6[P_2W_{17}O_{63}(AsC_6H_5)_2]$ (3)

Phenylarsonic acid (50.5mg, 0.250mmol) was dissolved in DMF (15ml) with stirring to give a colourless solution. To this was added $K_{10}[P_2W_{17}O_{61}]$ (0.5g, 0.110mmol) followed by 12M HCl (49μL, 0.588mmol) giving a pale turbid solution. The solution was then heated at 90°C for 16hrs after which it had become a cloudy off-white suspension. This was cooled to room temperature, filtered, and to this was added dry N-tetraethylammonium bromide (0.211g, 1mmol) and the mixture was stirred for 1hr. The solvent was then removed *in vacuo*, giving a pale blue powder, and the solid was redissolved in the minimum amount of anhydrous DMF and precipitated by the addition of a large excess of MeCN. The suspension was then centrifuged, the supernatant discarded, and then the powder was sonicated in a large excess of MeCN. This was again centrifuged, the supernatant discarded and the solvent was removed *in vacuo* to give the product as a pale blue powder. (0.332g, 66%). Single crystals of **3** could be grown by dissolution of 20mg of powder in DMSO, followed by vapour diffusion of EtOAc into the solution to yield single crystals of **3** after *ca.* 2 weeks (see crystallographic section for details). ^{1}H NMR (DMSO-d_6, 500MHz, ppm) δ 8.10-8.03 (m, 4H), 7.78-7.72 (m, 2H), 7.71-7.65 (m, 4H), 3.23 (q, J=7.2Hz, 48H), 1.18 (tt, J=7.3, 1.9Hz, 72H). ^{31}P NMR (DMSO-d_6, 500MHz, ppm) δ -11.31, -13.26. ATR-IR (cm^{-1}): 2978 (w,ν_{C-H}), 1739 (w, $\nu_{As-O\ overtone}$), 1483 (m,ν_{C-H}), 1391 (m,ν_{C-H}), 1085 (m, ν_{P-O}), 952 (s, $\nu_{W=O}$), 915 (s, ν_{P-O}), 773 (vs, ν_{W-O-W}).

The TBA salt (**8**) of this anion was prepared in a similar fashion, substituting TEA for equimolar equivalents of TBA. During the work-up procedure, DMF was substituted for MeCN and MeCN was substituted for $CHCl_3$. ^{1}H NMR (DMSO-d_6, 500MHz, ppm) δ 8.25-8.20 (m, 4H), 7.77-7.66 (m, 6H), 3.18-3.08 (m, 48H), 1.65 (tt, J=8.2, 6Hz, 72H), 1.41 (h, J=7.4, 48H) 1.01 (t, J=7.4, 72H). ^{31}P NMR (DMSO-d_6, 500MHz, ppm) δ -11.12, -13.06.

Synthesis of $(TEA)_6[P_2W_{17}O_{63}(AsC_6H_4NH_2)_2]$ (4)

p-Arsanilic acid (54.3mg, 0.250mmol) was dissolved in DMF (15ml) with stirring to give a colourless solution. To this was added $K_{10}[P_2W_{17}O_{61}]$ (0.5g, 0.110mmol) followed by 12M HCl (49µL, 0.588mmol) which formed a turbid orange solution. The solution was then heated at 90°C for 16hrs after which it had become a cloudy orange-red suspension. This was cooled to room temperature, filtered and to this was added dry N-tetraethylammonium bromide (0.211g, 1mmol) and the mixture was stirred for 1hr. The solvent was then removed *in vacuo*, giving an orange powder, and the solid was redissolved in the minimum amount of anhydrous DMF and precipitated by the addition of a large excess of MeCN. The suspension was then centrifuged, the supernatant discarded, and then the powder was sonicated in a large excess of MeCN. This was again centrifuged, the supernatant discarded and the solvent was removed *in vacuo* to give the product as an orange powder. (0.354g, 71%). ^1H NMR (DMSO-d_6, 500MHz, ppm) δ 7.66 (d, J= 9.0Hz, 4H), 6.70 (d, J=8.9Hz, 4H), 5.97 (s, 4H), 3.23 (q, J=7.2Hz, 48H), 1.18 (tt, J=7.5, 1.7Hz, 72H). ^{31}P NMR (DMSO-d_6, 500MHz, ppm) δ -11.34, -13.36. ATR-IR (cm^{-1}): 3353 (w,ν_{N-H}), 2988 (w,ν_{C-H}), 1739 (w, $\nu_{As-O\ overtone}$), 1593 (w,ν_{N-H}), 1482 (m,ν_{C-H}), 1391 (m,ν_{C-H}), 1085 (m, ν_{P-O}), 953 (s, $\nu_{W=O}$), 914 (s, ν_{P-O}), 775 (vs, ν_{W-O-W}).

Synthesis of $(TEA)_6[P_2W_{17}O_{63}(AsC_6H_4NO_2)_2]$ (5)

p-Nitarsone (61.8mg, 0.250mmol) was dissolved in DMF (15ml) with stirring to give a colourless solution. To this was added $K_{10}[P_2W_{17}O_{61}]$ (0.5g, 0.110mmol) followed by 12M HCl (49µL, 0.588mmol) which formed a pale turbid solution. The solution was then heated at 90°C for 16hrs after which it had become a cloudy off-white suspension. This was cooled to room temperature, filtered and to this was added dry N-tetraethylammonium bromide (0.211g, 1mmol) and the mixture was stirred for 1hr. The solvent was then removed *in vacuo*, giving a blue powder, and the solid was redissolved in the minimum amount of anhydrous DMF and precipitated by the addition of a large excess of $CHCl_3$/MeCN (3:1). The suspension was then centrifuged, the supernatant discarded, and then the powder was sonicated in a large excess of MeCN. This was again centrifuged, the supernatant discarded and the solvent was removed *in vacuo* to give the product as a blue powder. (0.224g, 45%).^1H NMR (DMSO-d_6, 500MHz, ppm) δ 8.55 (d, J= 9.0Hz, 4H), 8.33 (d, J=8.9Hz, 4H), 3.23 (q, J=7.2Hz, 48H), 1.18 (tt, J=7.2, 1.9Hz, 72H). ^{31}P NMR (DMSO-d_6, 500MHz, ppm) δ -11.32, -13.15. ATR-IR (cm^{-1}): 2985 (w,ν_{C-H}), 1739 (w, $\nu_{As-O\ overtone}$), 1663 (m,ν_{N-O}), 1527 (m,ν_{N-O})1483 (m,ν_{C-H}), 1391 (m,ν_{C-H}), 1350 (m,ν_{C-N}) 1086 (m, ν_{P-O}), 955 (s, $\nu_{W=O}$), 916 (s, ν_{P-O}), 780 (vs, ν_{W-O-W}).

Mass Spectrometry

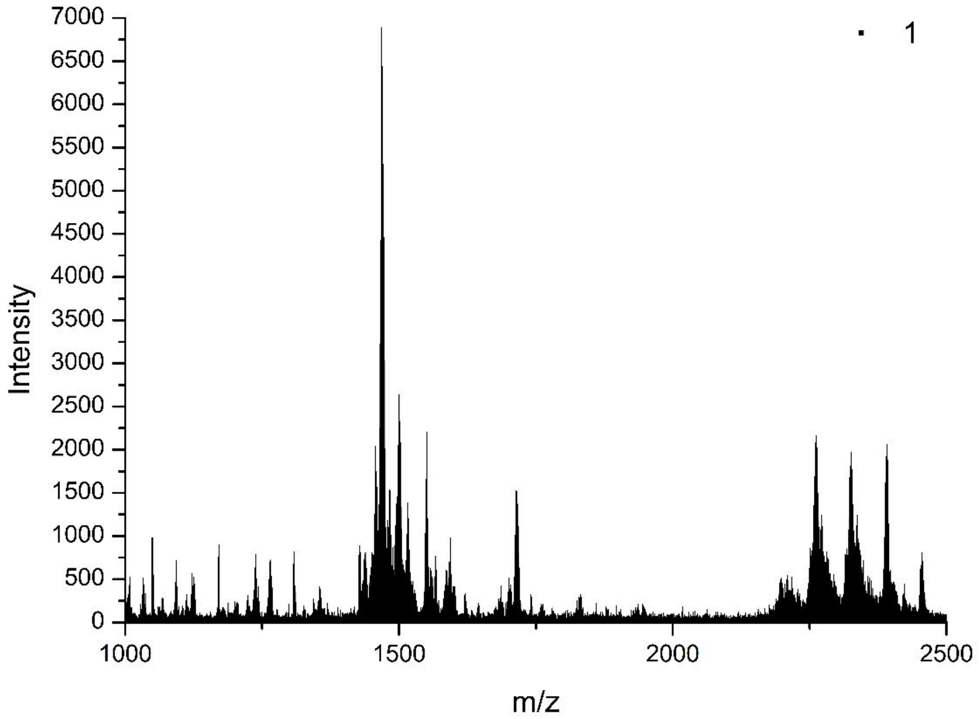

Figure S1. Negative mode ESI-MS spectrum of compound **1** in the m/z range 1000-2500

m/z(obs)	Z	Assignment	m/z(calc)
2455.25	2	$(TEA_4[P_2W_{17}O_{62}(SiC_6H_5)_2])$	2455.24
2390.66	2	$(TEA_3H_1[P_2W_{17}O_{62}(SiC_6H_5)_2])$	2390.66
2326.09	2	$(TEA_2H_2[P_2W_{17}O_{62}(SiC_6H_5)_2])$	2326.09
2262.51	2	$(TEA_1H_3[P_2W_{17}O_{62}(SiC_6H_5)_2])$	2261.51
1593.44	3	$(TEA_3\ [P_2W_{17}O_{62}(SiC_6H_5)_2])$	1593.44
1550.38	3	$(TEA_2H_1[P_2W_{17}O_{62}(SiC_6H_5)_2])$	1550.38

Table S1. Selected mass spectrometry peak assignemtns for compound **1.**

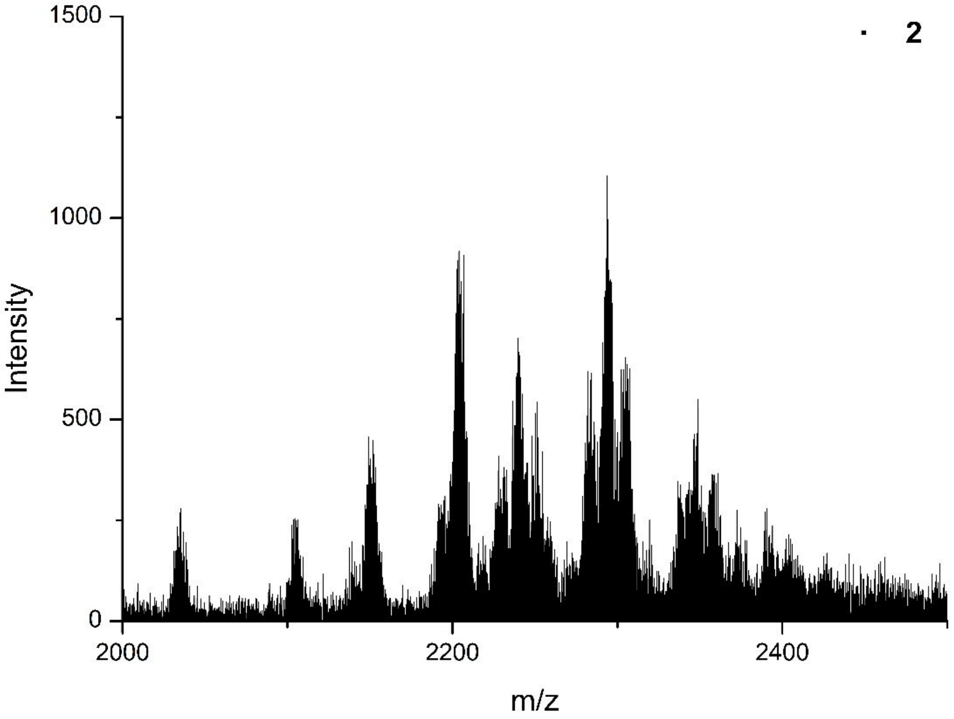

Figure S2. Negative mode ESI-MS spectrum of compound **2** in the m/z range 2000-2500

m/z(obs)	Z	Assignment	m/z(calc)
2359.03	2	$(TEA_2Na_2[P_2W_{17}O_{61}(POC_6H_5)_2])$	2359.06
2348.04	2	$(TEA_2Na_1H_1[P_2W_{17}O_{61}(POC_6H_5)_2])$	2348.07
2305.45	2	$(TEA_1Na_3[P_2W_{17}O_{61}(POC_6H_5)_2])$	2305.48
2293.96	2	$(TEA_1Na_2H_1[P_2W_{17}O_{61}(POC_6H_5)_2])$	2293.98
2283.46	2	$(TEA_1Na_1H_2[P_2W_{17}O_{61}(POC_6H_5)_2])$	2283.49
2251.88	2	$(Na_4[P_2W_{17}O_{61}(POC_6H_5)_2])$	2251.89
2240.88	2	$(Na_3H_1[P_2W_{17}O_{61}(POC_6H_5)_2])$	2240.90

2229.88	2	$(Na_2H_2[P_2W_{17}O_{61}(POC_6H_5)_2])$	2229.91

Table S2. Selected mass spectrometry peak assignemtns for compound **2.**

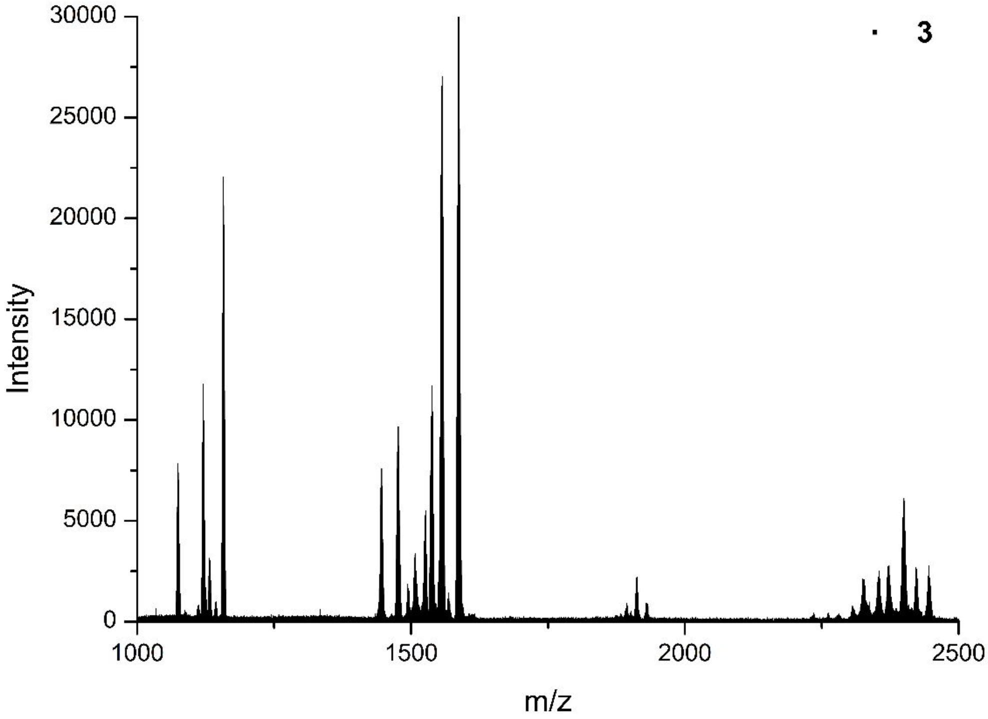

Figure S3. Negative mode ESI-MS spectrum of compound **3** in the m/z range 1000-2500

m/z(obs)	Z	Assignment	m/z(calc)
2445.64	2	$(TEA_3H_1[P_2W_{17}O_{61}(AsOC_6H_5)_2])$	2445.60
2422.34	4	$(TEA_5K_1[(P_2W_{17}O_{61}(AsOC_6H_5)_2)_2])$	2422.30
2400.05	2	$(TEA_2H_1K_1[P_2W_{17}O_{61}(AsOC_6H_5)_2])$	2400.00
2373.06	2	$(TEA_1K_3[P_2W_{17}O_{61}(AsOC_6H_5)_2])$	2373.38
2354.49	2	$(TEA_1H_1K_2[P_2W_{17}O_{61}(AsOC_6H_5)_2])$	2354.45
2326.96	2	$(TEA_1H_2Na_1[P_2W_{17}O_{61}(AsOC_6H_5)_2])$	2326.94
1930.29	5	$(TEA_5H_2[(P_2W_{17}O_{61}(AsOC_6H_5)_2)_2])$	1930.25
1912.05	5	$(TEA_4H_1K_2[(P_2W_{17}O_{61}(AsOC_6H_5)_2)_2])$	1912.01
1893.81	5	$(TEA_3H_2K_2[(P_2W_{17}O_{61}(AsOC_6H_5)_2)_2])$	1893.77
1586.71	3	$(TEA_2H_1[P_2W_{17}O_{61}(AsOC_6H_5)_2])$	1586.68

1556.64	3	$(TEA_1H_1K_1[P_2W_{17}O_{61}(AsOC_6H_5)_2])$	1556.62
1538.99	3	$(K_3[P_2W_{17}O_{61}(AsOC_6H_5)_2])$	1538.87
1526.24	3	$(H_1K_2[P_2W_{17}O_{61}(AsOC_6H_5)_2])$	1526.22
1507.93	3	$(H_1Na_2[P_2W_{17}O_{61}(AsOC_6H_5)_2])$	1507.91
1157.49	4	$(TEA_1H_1[P_2W_{17}O_{61}(AsOC_6H_5)_2])$	1157.47

Table S3. Selected mass spectrometry peak assignemtns for compound **3.**

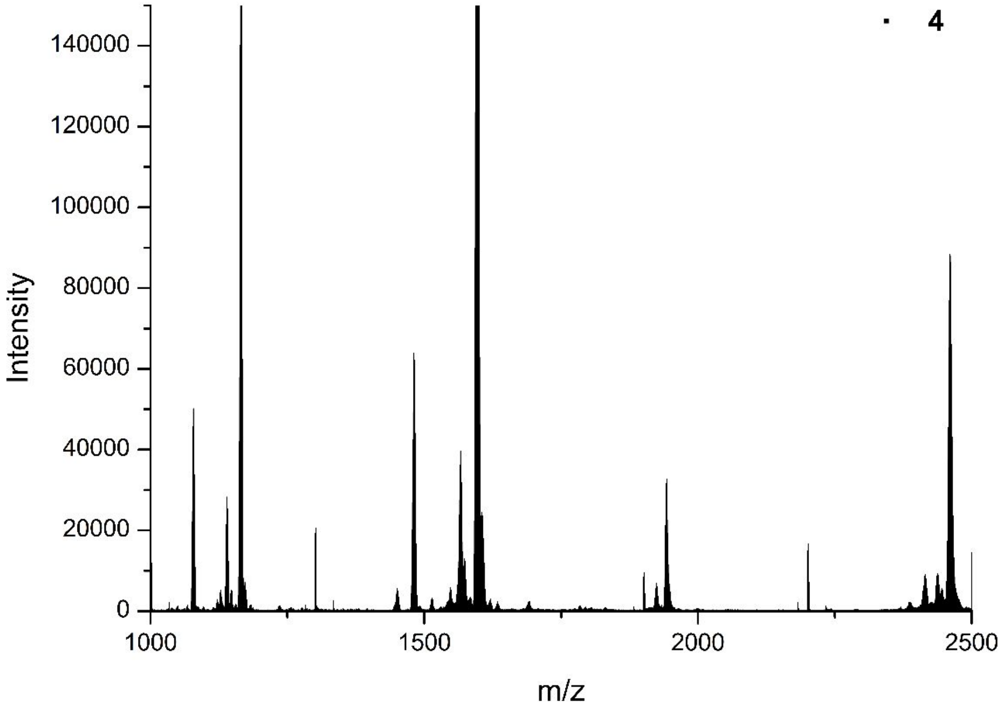

Figure S4. Negative mode ESI-MS spectrum of compound **4** in the m/z range 1000-2500

Table S4. Selected mass spectrometry peak assignemtns for compound **4.**

m/z(obs)	Z	Assignment	m/z(calc)
2460.63	2	$(TEA_3H_1[P_2W_{17}O_{61}(AsOC_6H_4NH_2)_2])$	2460.62
1942.06	5	$(TEA_5H_1[(P_2W_{17}O_{61}(AsOC_6H_4NH_2)_2)_2])$	1942.06
1923.83	5	$(TEA_4H_1K_1[(P_2W_{17}O_{61}(AsOC_6H_4NH_2)_2)_2])$	1923.82
1596.69	3	$(TEA_2H_1[P_2W_{17}O_{61}(AsOC_6H_4NH_2)_2])$	1596.69
1566.63	3	$(TEA_1H_1K_1[P_2W_{17}O_{61}(AsOC_6H_4NH_2)_2])$	1566.62
1164.98	4	$(TEA_1H_1[P_2W_{17}O_{61}(AsOC_6H_4NH_2)_2])$	1164.98

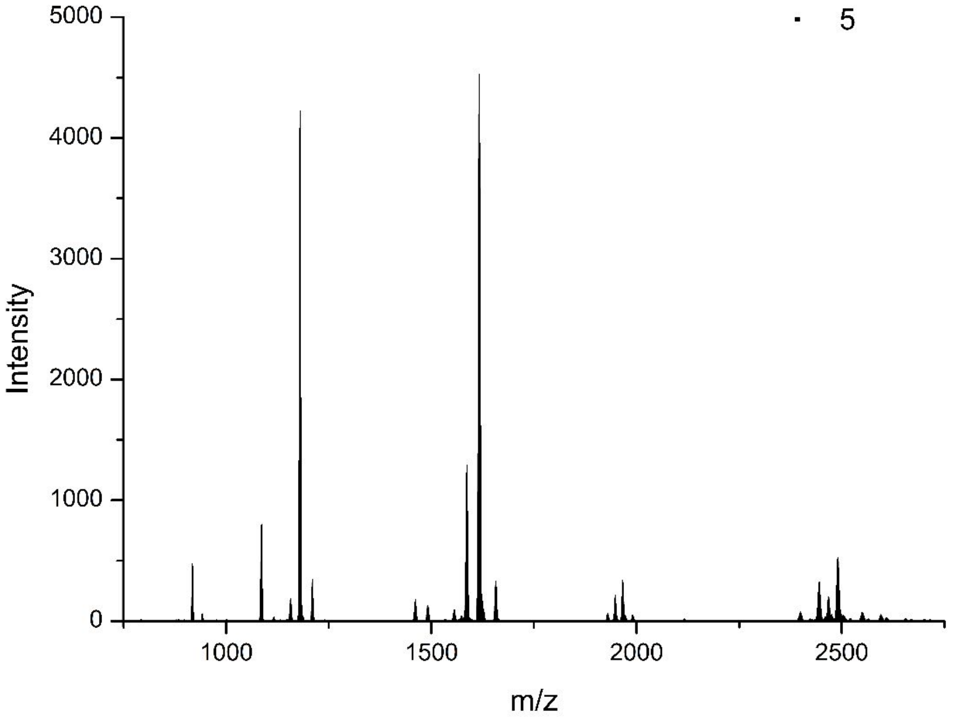

Figure S5. Negative mode ESI-MS spectrum of compound **5** in the m/z range 1000-2500

Table S5. Selected mass spectrometry peak assignemtns for compound **5.**

m/z(obs)	Z	Assignment	m/z(calc)
2490.59	2	$(TEA_3H_1[P_2W_{17}O_{61}(AsOC_6H_4NO_2)_2])$	2490.59
2467.79	4	$(TEA_5H_2K_1[P_2W_{17}O_{61}(AsOC_6H_4NO_2)_2]_2)$	2467.80
2444.99	2	$(TEA_2H_1K_1[P_2W_{17}O_{61}(AsOC_6H_4NO_2)_2])$	2444.99
2399.39	2	$(TEA_1H_1K_2[P_2W_{17}O_{61}(AsOC_6H_4NO_2)_2])$	2399.40

1966.24	5	$(TEA_5H_2[(P_2W_{17}O_{61}(AsOC_6H_4NO_2)_2)_2])$	1966.24
1950.00	5	$(TEA_4K_1H_2[(P_2W_{17}O_{61}(AsOC_6H_4NO_2)_2)_2])$	1950.00
1929.96	5	$(TEA_3K_2H_2[(P_2W_{17}O_{61}(AsOC_6H_4NO_2)_2)_2])$	1929.97
1617.01	3	$(TEA_2H_1[(P_2W_{17}O_{61}(AsOC_6H_4NO_2)_2)])$	1617.00
1586.61	3	$(TEA_1H_1K_1[P_2W_{17}O_{61}(AsOC_6H_4NO_2)_2])$	1586.61
1179.96	4	$(TEA_1H_1[P_2W_{17}O_{61}(AsOC_6H_4NO_2)_2])$	1179.97
917.54	5	$(H_1[P_2W_{17}O_{61}(AsOC_6H_4NO_2)_2])$	917.54

X-ray Crystallography

A single crystal was selected and mounted using Fomblin® (YR-1800 perfluoropolyether oil) on a polymer-tipped MiTeGen MicroMountTM and cooled rapidly to 120 K in a stream of cold N2 using an Oxford Cryosystems open flow cryostat.[4] Single crystal X-ray diffraction data were collected on an Oxford Diffraction SuperNova Duo diffractometer (Atlas CCD area detector, mirror-monochromated Cu-Kα radiation source; λ = 1.54184 Å, ω scans). Cell parameters were refined from the observed positions of all strong reflections and absorption corrections were applied using a Gaussian numerical method with beam profile correction (CrysAlisPro).[5] The structure was solved within Olex2[6] by dual space iterative methods (SHELXT)[7] and all non-hydrogen atoms refined by full-matrix least-squares on all unique F2 values with anisotropic displacement parameters (SHELXL).[8] Hydrogen atoms were refined

with constrained geometries and riding thermal parameters. PLATON SQUEEZE was applied to the data to remove scattering contributions from several disordered cations and solvent residues which could not be modelled as discrete sites.[9] Structures were checked with checkCIF.[10] CCDC-2222644 contains the supplementary data for these compounds. These data can be obtained free of charge from The Cambridge Crystallographic Data Centre via www.ccdc.cam.ac.uk/data_request/cif.

The structure was found to be compositionally disordered: the major component is an organoarsenic hybrid dawson polyoxotungstate which is co-crystallised with a minor component of the parent Dawson polyoxotungstate. The two species are sited such that their core structures share many atom positions with only atoms close to the site of functionalisation differing between the two species. The occupancies of the differentiated atoms of the two species are refined and constrained to sum to unity with the major (hybrid) and minor (plenary) components having values of 0.84(1) and 0.16(1) respectively.

Geometric similarity restraints have been applied to chemically equivalent W-O distances of atoms in the minor (plenary) to reflect the three-fold symmetry of the anion. These restraints were particularly necessary for the oxygen and tungsten atoms overlapping with the disordered parts of the structure (SADI). The phenyl rings are restrained to have planar geometry (FLAT) and have mirror symmetry (SADI). The C-N and C-C bonds of the two tetraethylammonium residues are restrained to have similar distances (SADI).

The polyoxotungstate oxygen atoms of the minor disorder component have been refined with isotropic displacement parameters and restrained to be similar to those of adjacent atoms of the major disorder component (SIMU). Rigid bond restraints have been applied to the carbon atoms of the phenyl ring moiety and tetraethylammonium residues (RIGU). The anisotropic displacement parameter of oxygen O26 is restrained to have more isotropic character. Tetraethylammonium carbon atom C7C is modelled with an isotropic displacement parameter which is fixed at a value of 0.2; it is likely this ethyl moiety features disorder which could not sensibly be modelled.

PLATON SQUEEZE was applied to the data to remove scattering contributions from several disordered cations and solvent residues which could not be modelled as discrete sites: 2299 electrons per unit cell were consistent with 1.5 tetraethylammonium cations, 2

dimethylsulphoxide solvent molecules and 2 ethyl acetate per asymmetric unit. These molecules have been included in the chemical formula and in all values derived from it. SQUEEZE also yielded a set of solvent-free diffraction intensities for use in the final cycles of refinement.

Table S6. Crystallographic details for compound **3**

**Sum Chemical Formula**	C10.14 H184.5 As1.69 N6 O74.85 P2 S4 W17.15
**Nominal Moiety Formula (inc. solvent and cations)**	C68 H152 As2 N8 O71 P2 S4 W17
**Mr (g mol-1)**	5925.40
**Crystal System**	Orthorhombic
**Space Group**	*Pnma*
a **(Å)**	35.384
b **(Å)**	25.697
c **(Å)**	15.325
α **(°)**	90
β **(°)**	90
γ **(°)**	90
**V ($Å^3$)**	13933.8
Z	4
**T (K)**	120
**$F_{(000)}$**	10951.0
**P_{calcd} ($g\ cm^{-3}$)**	2.825

μ(Mo$_{K\alpha}$) (mm^{-1})	27.406
Rflns. (measd.)	167022
Rflns. (uniq.)	14493
No. params.	598
R1 (I > 2σ(I))	0.0694
wR2 (all)	0.1708
S	1.069

Figure S6 & Table S7. Comparison of the bonding environment around arsenic in **3** with its phosphorous analogue and related organoarsenic phosphotungstate $[(C_6H_5AsO)_4P_8W_{48}O_{184}]^{32-}$

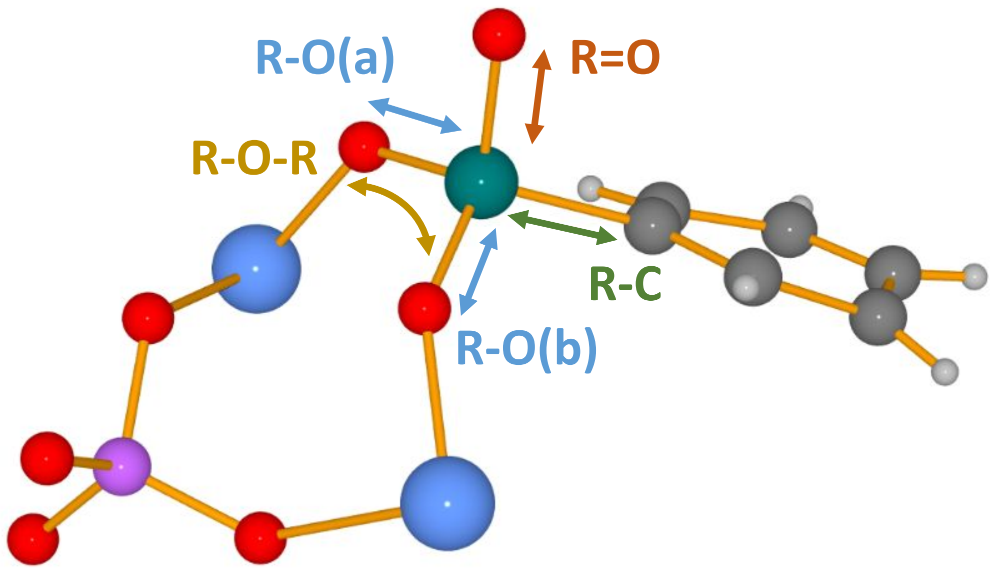

	3	$[P_2W_{17}O_{61}(POC_6H_5)_2]^{6-}$ [11]	$[(C_6H_5AsO)_4P_8W_{48}O_{184}]^{32-}$ [12]
R=O	1.667Å	1.525Å	1.662Å
R-O(a)	1.676Å	1.536Å	1.683Å
R-O(b)	1.701Å	1.542Å	1.718Å
R-C	1.900Å	1.790Å	1.890Å
R-O-R	111.0°	109.8°	102.6°

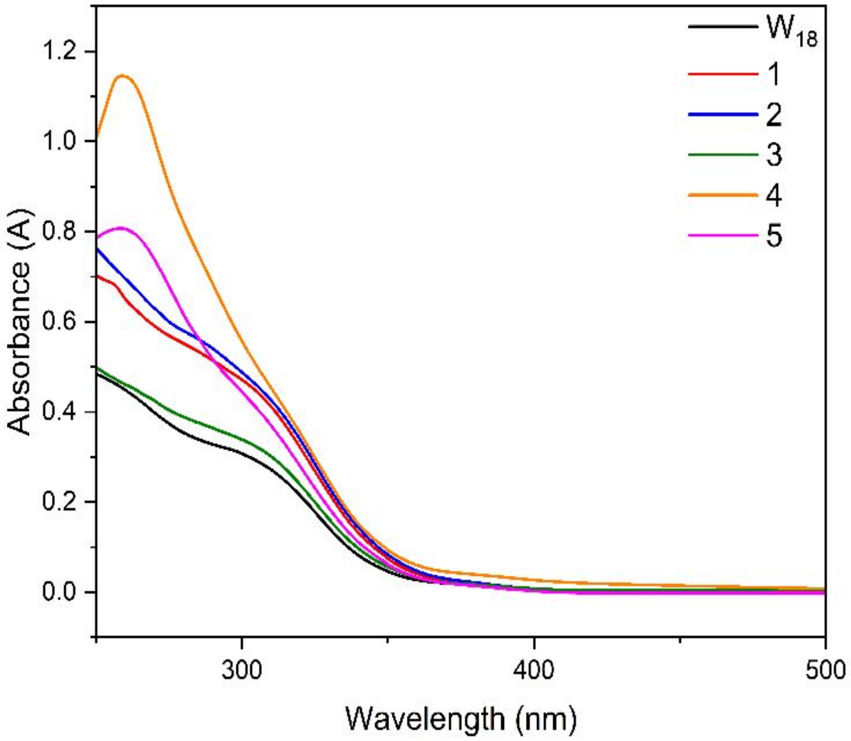

Figure S7. UV-vis absorption spectrum of compounds **1-5** in acetonitrile at 12.5 μM

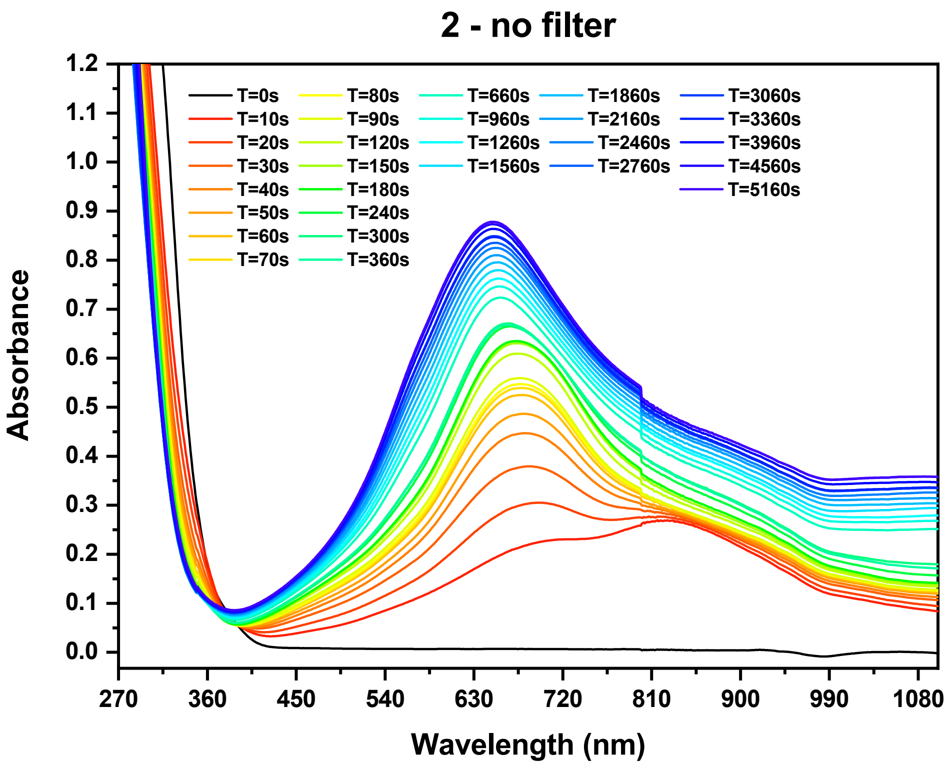

Figure S8. UV-vis absorption spectrum showing the photoreduction of **2** in DMF over time without a filter.

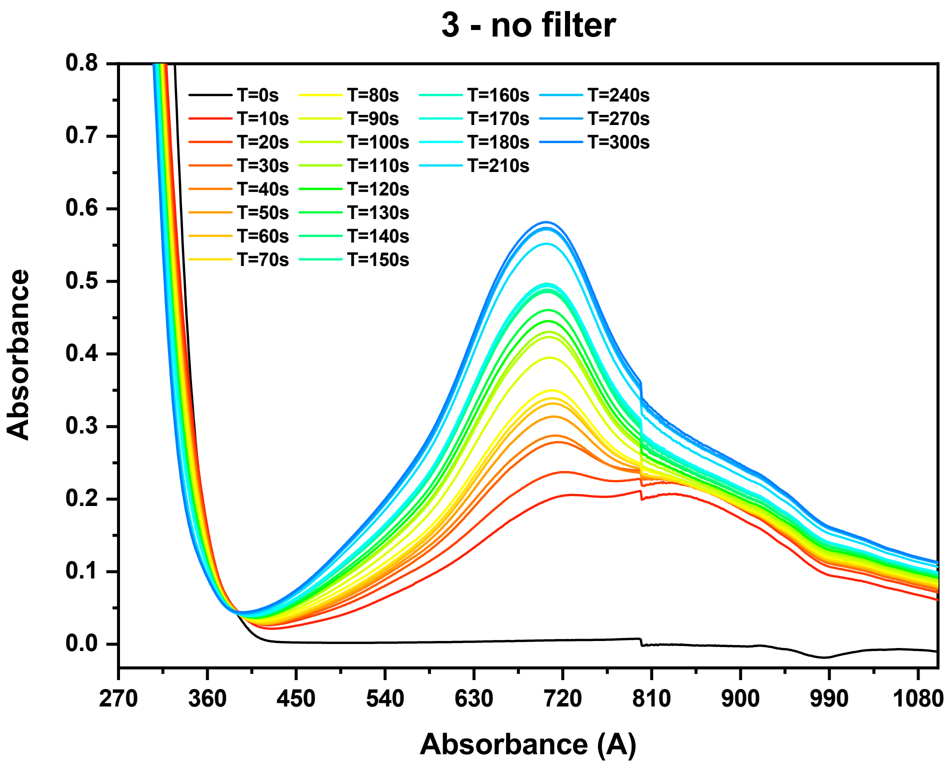

Figure S9. UV-vis absorption spectrum showing the photoreduction of **3** in DMF over time without a filter.

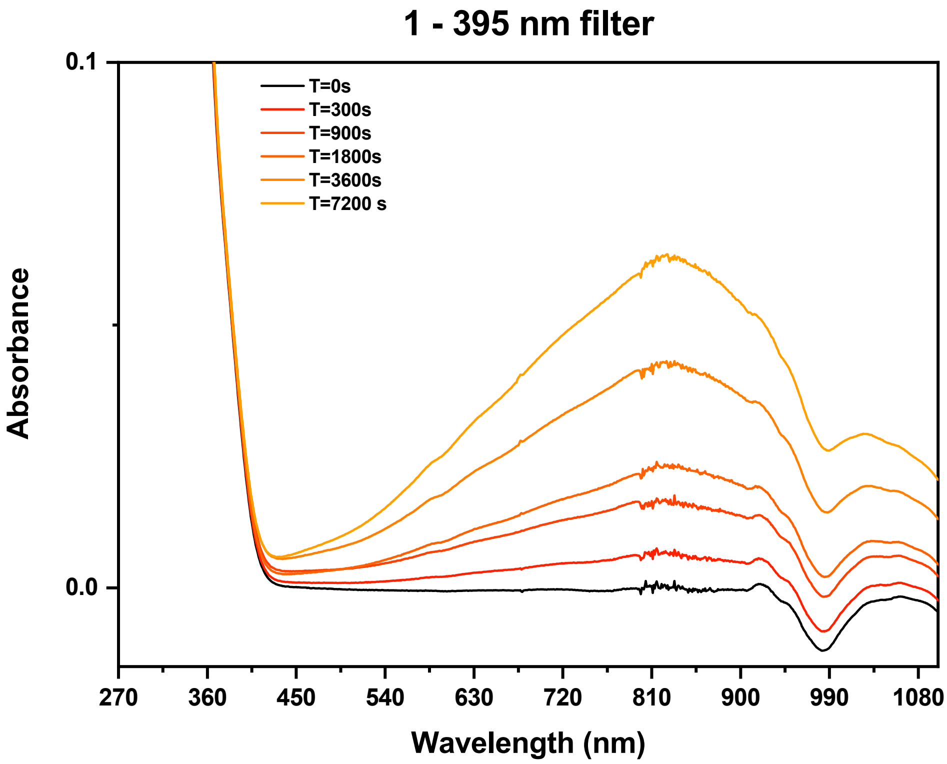

Figure S10. UV-vis absorption spectrum showing the photoreduction of **1** in DMF over time using a 395 nm filter.

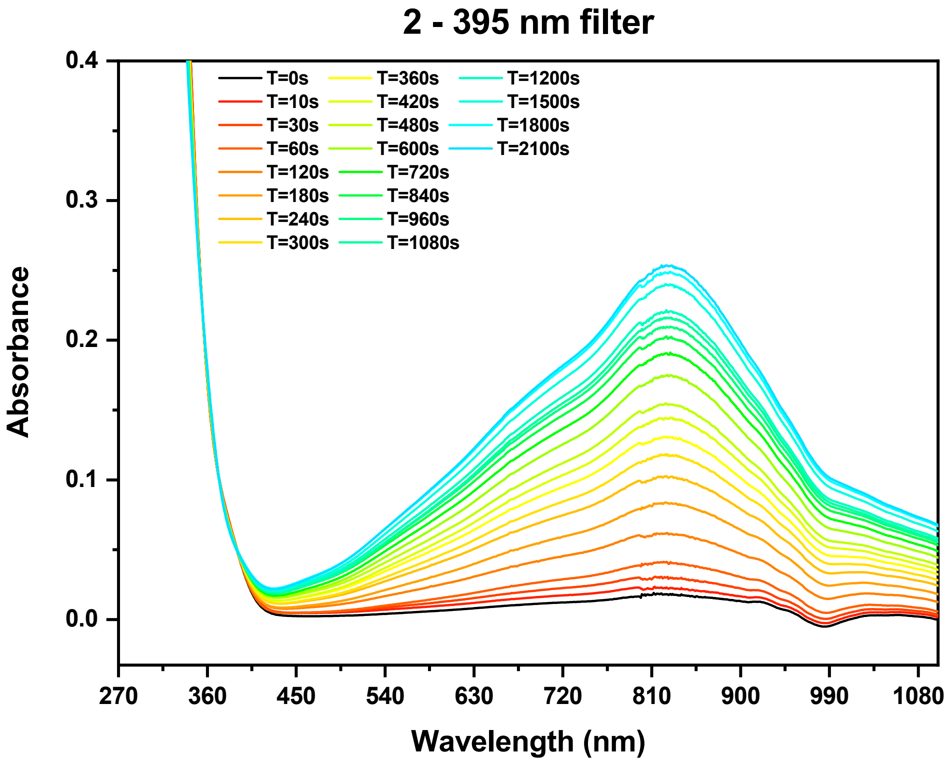

Figure S11. UV-vis absorption spectrum showing the photoreduction of **2** in DMF over time using a 395 nm filter.

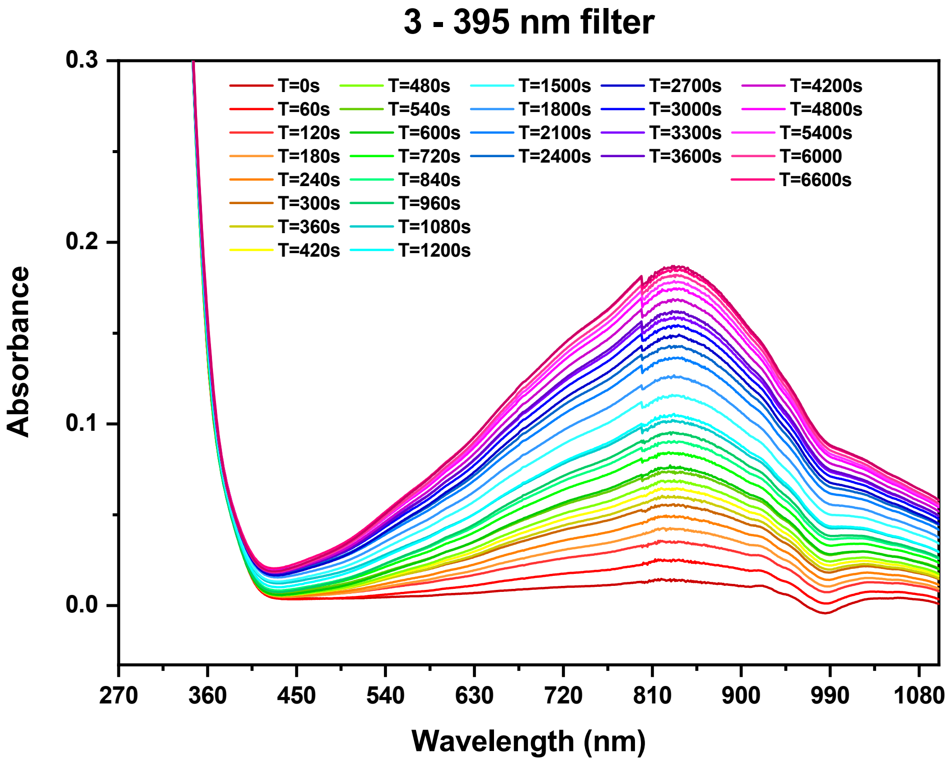

Figure S12. UV-vis absorption spectrum showing the photoreduction of **3** in DMF over time using a 395 nm filter.

References

1. Becke, A. D. In *Density Functional Calculations of Molecular Bond Energies*, Density Matrices and Density Functionals, Dordrecht, 1987//; Erdahl, R.; Smith, V. H., Eds. Springer Netherlands: Dordrecht, 1987; pp 443-455.
2. Ermler, W. C.; Ross, R. B.; Christiansen, P. A., Ab initio relativistic effective potentials with spin-orbit operators. VI. Fr through Pu. *International Journal of Quantum Chemistry* **1991,** *40* (6), 829-846.
3. Shao, Y.; Gan, Z.; Epifanovsky, E.; Gilbert, A. T. B.; Wormit, M.; Kussmann, J.; Lange, A. W.; Behn, A.; Deng, J.; Feng, X.; Ghosh, D.; Goldey, M.; Horn, P. R.; Jacobson, L. D.; Kaliman, I.; Khaliullin, R. Z.; Kuś, T.; Landau, A.; Liu, J.; Proynov, E. I.; Rhee, Y. M.; Richard, R. M.; Rohrdanz, M. A.; Steele, R. P.; Sundstrom, E. J.; Woodcock, H. L.; Zimmerman, P. M.; Zuev, D.; Albrecht, B.; Alguire, E.; Austin, B.; Beran, G. J. O.; Bernard, Y. A.; Berquist, E.; Brandhorst, K.; Bravaya, K. B.; Brown, S. T.; Casanova, D.; Chang, C.-M.; Chen, Y.; Chien, S. H.; Closser, K. D.; Crittenden, D. L.; Diedenhofen, M.; DiStasio, R. A.; Do, H.; Dutoi, A. D.; Edgar, R. G.; Fatehi, S.; Fusti-Molnar, L.; Ghysels, A.; Golubeva-Zadorozhnaya, A.; Gomes, J.; Hanson-Heine, M. W. D.; Harbach, P. H. P.; Hauser, A. W.; Hohenstein, E. G.; Holden, Z. C.; Jagau, T.-C.; Ji, H.; Kaduk, B.; Khistyaev, K.; Kim, J.; Kim, J.; King, R. A.; Klunzinger, P.; Kosenkov, D.; Kowalczyk, T.; Krauter, C. M.; Lao, K. U.; Laurent, A. D.; Lawler, K. V.; Levchenko, S. V.; Lin, C. Y.; Liu, F.; Livshits, E.; Lochan, R. C.; Luenser, A.; Manohar, P.; Manzer, S. F.; Mao, S.-P.; Mardirossian, N.; Marenich, A. V.; Maurer, S. A.; Mayhall, N. J.; Neuscamman, E.; Oana, C. M.; Olivares-Amaya, R.; O'Neill, D. P.; Parkhill, J. A.; Perrine, T. M.; Peverati, R.; Prociuk, A.; Rehn, D. R.; Rosta, E.; Russ, N. J.; Sharada, S. M.; Sharma, S.; Small, D. W.; Sodt, A.; Stein, T.; Stück, D.; Su, Y.-C.; Thom, A. J. W.; Tsuchimochi, T.; Vanovschi, V.; Vogt,

L.; Vydrov, O.; Wang, T.; Watson, M. A.; Wenzel, J.; White, A.; Williams, C. F.; Yang, J.; Yeganeh, S.; Yost, S. R.; You, Z.-Q.; Zhang, I. Y.; Zhang, X.; Zhao, Y.; Brooks, B. R.; Chan, G. K. L.; Chipman, D. M.; Cramer, C. J.; Goddard, W. A.; Gordon, M. S.; Hehre, W. J.; Klamt, A.; Schaefer, H. F.; Schmidt, M. W.; Sherrill, C. D.; Truhlar, D. G.; Warshel, A.; Xu, X.; Aspuru-Guzik, A.; Baer, R.; Bell, A. T.; Besley, N. A.; Chai, J.-D.; Dreuw, A.; Dunietz, B. D.; Furlani, T. R.; Gwaltney, S. R.; Hsu, C.-P.; Jung, Y.; Kong, J.; Lambrecht, D. S.; Liang, W.; Ochsenfeld, C.; Rassolov, V. A.; Slipchenko, L. V.; Subotnik, J. E.; Van Voorhis, T.; Herbert, J. M.; Krylov, A. I.; Gill, P. M. W.; Head-Gordon, M., Advances in molecular quantum chemistry contained in the Q-Chem 4 program package. *Molecular Physics* **2015,** *113* (2), 184-215.
4. Cosier, J.; Glazer, A. M., A nitrogen-gas-stream cryostat for general X-ray diffraction studies. *Journal of Applied Crystallography* **1986,** *19* (2), 105-107.
5. Oxford Rigaku Diffraction, *CrysAlisPro Software system version 1.171.40.45a*, Rigaku Corporation: Oxford, UK, 2018.
6. Dolomanov, O. V.; Bourhis, L. J.; Gildea, R. J.; Howard, J. A. K.; Puschmann, H., OLEX2: a complete structure solution, refinement and analysis program. *Journal of Applied Crystallography* **2009,** *42* (2), 339-341.
7. Sheldrick, G., SHELXT - Integrated space-group and crystal-structure determination. *Acta Crystallographica Section A* **2015,** *71* (1), 3-8.
8. Sheldrick, G., Crystal structure refinement with SHELXL. *Acta Crystallographica Section C* **2015,** *71* (1), 3-8.
9. Spek, A., PLATON SQUEEZE: a tool for the calculation of the disordered solvent contribution to the calculated structure factors. *Acta Crystallographica Section C* **2015,** *71* (1), 9-18.
10. “CheckCIF,” can be found under http://checkcif.iucr.org.
11. Fujimoto, S.; Cameron, J. M.; Wei, R.-J.; Kastner, K.; Robinson, D.; Sans, V.; Newton, G. N.; Oshio, H., A Simple Approach to the Visible-Light Photoactivation of Molecular Metal Oxides. *Inorganic Chemistry* **2017,** *56* (20), 12169-12177.
12. Yi, X.; Izarova, N. V.; Kögerler, P., Organoarsonate Functionalization of Heteropolyoxotungstates. *Inorganic Chemistry* **2017,** *56* (22), 13822-13828.
